# Novel and Transgressive Salinity Tolerance in Recombinant Inbred Lines of Rice Created by Physiological Coupling-Uncoupling and Network Rewiring Effects

**DOI:** 10.3389/fpls.2021.615277

**Published:** 2021-02-23

**Authors:** Isaiah C. M. Pabuayon, Ai Kitazumi, Kevin R. Cushman, Rakesh Kumar Singh, Glenn B. Gregorio, Balpreet Dhatt, Masoud Zabet-Moghaddam, Harkamal Walia, Benildo G. de los Reyes

**Affiliations:** ^1^Department of Plant and Soil Science, Texas Tech University, Lubbock, TX, United States; ^2^International Rice Research Institute, Los Baños, Philippines; ^3^Department of Agronomy and Horticulture, University of Nebraska-Lincoln, Lincoln, NE, United States; ^4^Center for Biotechnology and Genomics, Texas Tech University, Lubbock, TX, United States

**Keywords:** genetic novelty, genetic network rewiring, salinity stress, transgressive segregation, physiological and biochemical synergy, Omnigenic Theory

## Abstract

The phenomenon of transgressive segregation, where a small minority of recombinants are outliers relative to the range of parental phenotypes, is commonly observed in plant breeding populations. While this phenomenon has been attributed to complementation and epistatic effects, the physiological and developmental synergism involved have not been fully illuminated by the QTL mapping approach alone, especially for stress-adaptive traits involving highly complex interactions. By systems-level profiling of the IR29 × Pokkali recombinant inbred population of rice, we addressed the hypothesis that novel salinity tolerance phenotypes are created by reconfigured physiological networks due to positive or negative coupling-uncoupling of developmental and physiological attributes of each parent. Real-time growth and hyperspectral profiling distinguished the transgressive individuals in terms of stress penalty to growth. Non-parental network signatures that led to either optimal or non-optimal integration of developmental with stress-related mechanisms were evident at the macro-physiological, biochemical, metabolic, and transcriptomic levels. Large positive net gain in super-tolerant progeny was due to ideal complementation of beneficial traits while shedding antagonistic traits. Super-sensitivity was explained by the stacking of multiple antagonistic traits and loss of major beneficial traits. The synergism uncovered by the phenomics approach in this study supports the modern views of the Omnigenic Theory, emphasizing the synergy or lack thereof between core and peripheral components. This study also supports a breeding paradigm rooted on genomic modeling from multi-dimensional genetic, physiological, and phenotypic profiles to create novel adaptive traits for new crop varieties of the 21st century.

## Introduction

Plant breeding during the Green Revolution created the modern cultivars of rice, maize, and wheat with superior yields under well-managed environments with ideal water and nutrient conditions. Because of climate change and natural resources deterioration, the challenges to the 21st century plant breeding have become even grander (de los Reyes, 2019). Developing the next generation of crops with minimal penalty to yield under marginalized environments is the new overarching goal. Key to this goal are new genetic variants and novel phenotypes that may not have been achieved in the past.

To create the much-needed genetic novelties, a non-reductionist approach is important in considering several critical questions. Has plant breeding fully exhausted the combining potential of exotic germplasm to create adaptive phenotypes that are more relevant today? With genomic biology, there is a strong optimism that strategic utilization of exotic germplasm could lead to further improvements in complex traits that define yield potential under marginal environments. Genomics-enabled selection allowed breeders to evaluate genetic gains through the inheritance of component QTL (Li et al., 2005; Lippman et al., 2007; Thomson et al., 2010). Stacking of QTL promises to create optimal combinations that maximize the additive and non-additive potentials of parents (deVicente and Tanksley, 1993; Eshed and Zamir, 1995; Tanksley and Nelson, 1996; Ashikari and Matsuoka, 2006; Kumar et al., 2018; Sandhu et al., 2018). Are further genetic gains on top of what had been achieved post-Green Revolution still possible? How can the power of genomics be used for additional genetic gains?

The questions presented should inspire critical thinking for integrating the classic phenomena in plant breeding with genomic biology toward the much-needed genetic novelties. Transgressive segregation is a good example of such phenomena, characterized by heritable variation across the progenies of genetically divergent parents where a small minority are beyond the parental range. It was proposed that transgressive phenotypes in breeding populations are similar to genetic novelties occurring in populations of natural hybrids, which is believed to nucleate adaptive speciation (Rieseberg et al., 1996, 2003; Dickinson et al., 2003; de los Reyes, 2019). Inspired by such evolutionary theory, the classic phenomenon of transgressive segregation must be re-envisioned as a possible means to achieve further genetic gains in adaptive potentials for the crops of the 21st century.

Epistatic interaction and complementation of additive alleles have been attributed to the superior or inferior attributes of transgressive segregants (Dittrich-Reed and Fitzpatrick, 2013). In rice for example, QTL complementation created transgressive developmental traits such as leaf tiller angle (Xu et al., 1998). However, while the interacting major-effect QTL explained to a significant extent the observed phenotypic variance, the range of transgressive phenotypes across the population appeared to be complex enough to ignore the hidden contributions from many other interacting loci with minute effects, which may not be fully discerned by QTL mapping or genome-wide association studies (GWAS) alone. Similarly, seminal studies in Arabidopsis identified major interacting loci that caused transgressive segregation for qualitative disease resistance (Koornneef et al., 2004; Kover et al., 2005; Staal et al., 2006). However, the number of interacting loci revealed by such qualitative traits may not be equally reflective of the complexity of physiological interactions when large numbers of major-effect and minor-effect components are involved, like in the case of stress-adaptive traits.

The recently proposed Omnigenic Theory opens an alternative view to explain the most critical interactions underlying transgressive phenotypes, when large number of physiological components are likely to be involved (Boyle et al., 2017). This theory defines quantitative traits in terms of both additive and non-additive contributions of few large-effect core loci and hundreds of minute-effect peripheral loci across the genome. We further hypothesized that the superiority or inferiority of transgressive segregants are due to ideal or non-ideal coupling-uncoupling of various compatible and incompatible biochemical, developmental, and physiological attributes from each parent encoded by the core and peripheral loci. These mechanisms lead to physiological gain or drag, determining positive or negative net gains in certain individuals (de los Reyes, 2019). Because of the many attributes that should be affected by the coupling-uncoupling mechanisms, it would take many generations of genome reshuffling to observe the network rewiring effects in rare recombinants.

To test the physiological coupling-uncoupling and network rewiring hypotheses, we employed a multi-tier approach to profile a well-characterized recombinant inbred population of rice developed by the International Rice Research Institute (IRRI) with the salt-sensitive IR29 (*Xian/Indica*) and salt-tolerant Pokkali (*Aus*) as parents (Bonilla et al., 2002; Gregorio et al., 2002; Walia et al., 2005; Singh et al., 2007; Thomson et al., 2010). The photoperiod-sensitive Pokkali has been historically used as donor of salinity tolerance in rice breeding (Moeljopawiro and Ikehashi, 1981; Sahi et al., 2006). We were particularly interested in exploring the full combining potential of this donor with a high-yielding *Xian/Indica* cultivar (IR29) beyond what has been revealed by QTL mapping, which attributed about 40% of phenotypic variance at early seedling stage (V1 to V4) to *Saltol* on chromosome-1 (Ren et al., 2005; Thomson et al., 2010).

The recombinant inbred line (RIL) FL478 has served as the non-photoperiod sensitive donor of *Saltol* for *Xian/Indica* cultivars (Islam et al., 2012; Huyen et al., 2013; Chattopadhyay et al., 2014; Bimpong et al., 2016; Waziri et al., 2016; Babu et al., 2017). While *Saltol* had a large effect at the seedling stage, by revealing its buried physiological synergies under severe salinity stress at the vegetative stage, this study sought to understand the mechanisms that maximize its impact toward a transgressive phenotype that may not have been revealed by QTL mapping alone. We describe here the multi-tier macro-physiological, biochemical, and molecular profiling of IR29 × Pokkali RILs at the population and individual levels. We scrutinized individuals representing each phenotypic class to redefine the critical physiological and developmental aspects of transgressive segregants. Results support the coupling-uncoupling and network rewiring hypotheses by revealing the hidden potentials of the sensitive parent IR29 toward positive complementation with *Saltol* and other physiological attributes from Pokkali. We also revealed the potential of Pokkali for physiological drags against other minor-effect components.

## Materials and Methods

### Phenotypic Evaluation of the RIL Population

The RILs of IR29 (*Xian/Indica*; salt-sensitive) × Pokkali (*Aus*; salt-tolerant) consisted of 123 individuals as the core QTL mapping population. Segregation for salinity tolerance was established earlier at IRRI based on Standard Evaluation Score (SES) and shoot Na^+^/K^+^ at early seedling (V1 to V4) stage for the fine-mapping of *Saltol* at an electrical conductivity (EC) level of 9 dS m^–1^ (Singh et al., 2007; Thomson et al., 2010). Electric conductivity is defined as the capacity of a medium to pass an electric current. It is used as a measure of salinity as it also reliably measures the ion content of the medium (Corwin and Yemoto, 2017). A comparative panel of 67 individuals representing the full range of SES and Na^+^/K^+^ was subjected to a multi-parameter replicated (*n* = 8) evaluation during an extended vegetative growth window (V4 to V12) in hydroponics at 30–35°C day, 24–26°C night, 20 to 30% RH, and 12-h photoperiod with 500 μmol m^–2^s^–1^ light intensity (Counce et al., 2000). Physiological parameters included SES, Na^+^/K^+^, electrolyte leakage index (ELI), peroxidase (POX) activity, lipid peroxidation (LP), and shoot biomass.

Fourteen day-old seedlings were transplanted to 0.6-gallon buckets with 1 g/L Peter’s Professional 20-20-20 Fertilizer (JR Peters Inc., Allentown, PA, United States) at pH 5.8, supplemented with 0.4 g L^–1^ FeSO_4_⋅7H_2_O. Plants were subjected to salinity stress at tillering stage (V4 to V12) with ∼120 mM NaCl (EC = 12 dS m^–1^). Samples for physiological assays, RNA extraction, and biomass measurements were collected at 0 hr (control) and after 24, 48, 72, and 144 h of stress. At least three biological replicates were sampled for each experiment described below.

### Biomass, Stomatal Conductance, and Standard Evaluation Score (SES)

Shoot fresh weight was measured from three plants at 0 and 144 h after stress. Tissue samples were oven-dried (50°C) for 5 days to determine the % biomass. Biomass ratio >1 indicates growth, while a ratio <1 indicates penalty. Stomatal conductance was measured by SC-1 Leaf Porometer (Meter Group Inc., Pullman, WA, United States) at 0 and 144 h and presented as control/stress ratio. At the end of stress treatments (17 days), the plant health status was rated visually using IRRI’s Standard Evaluation System (SES) based on a ranking of 1 to 9, with a higher score indicating a more severe stress injury (Gregorio et al., 1997). To make the SES scores directly proportional to the other phenotypic scores, it was inverted to a scale of 1 to 10, with 1 having the worst performance (susceptible) and 10 having the best performance (tolerant).

### Electrolyte Leakage Index

Leaf Electrolyte Leakage Index (ELI) was used as a function of cellular injury from osmotic stress as well as ion uptake (Ballou et al., 2007; de los Reyes et al., 2013). Leaf disks were sampled from individual plants (*n* = 5) and placed in 5 mL ultrapure water (18 megaOhms). Electrical conductivity (EC) was measured with a conductivity meter (Thermo Scientific, Waltham, MA, United States) after leaching in solution (stress EL). Total tissue electrolyte was measured after boiling at 95°C. Electrolyte leakage was calculated as: *EL_sample_ = EC_boiled_/EC_unboiled_ × 100*. ELI was calculated with the following equation and expressed as a mean of ELI (*n* = 5): *ELI = EL_144 h_/EL_0 h_* × *100*.

### Peroxidase Activity Assay

Total peroxidase activity (POX) was measured with the Amplex^®^ Red Hydrogen Peroxide/Peroxidase Assay Kit according to manufacturer’s instructions (Invitrogen, Carlsbad, CA, United States). Pulverized leaves were homogenized in 20 mM sodium phosphate buffer (pH 6.5) and centrifuged at 9,000 × *g* for 10 min at 4°C. Samples were reacted with Amplex^®^ Red mix in the dark for 30 min, and absorbance at 560 nm was presented as means (*n* = 3). POX was based on a standard curve of pure horseradish peroxidase at a range of 0 to 2 mU mL^–1^.

### Lipid Peroxidation Assay

Leaf samples (*n* = 3) were pulverized in liquid nitrogen, homogenized with 0.1% trichloroacetic acid (TCA), and centrifuged at 10,000 × *g* for 15 min (Jambunathan, 2010). Supernatant was combined with 20% TCA and 0.5% thiobarbituric acid (TBA) and incubated at 95°C for 30 min. Absorbance was measured at 530 and 600 nm, with A_600_ as background absorbance against A_532_. Values were used to determine the malondialdehyde (MDA) content as estimate of broken lipid membranes reacting with TBA. MDA content was based on Beer-Lambert equation: *C* = *A*/ε × *l*, where A, differences in absorbance at 530 and 600 nm; ε, extinction coefficient of 155 mM^–1^cm^–1^; *l*, length (cm) of light path; and *C*, content (mM) of MDA.

### Na^+^ and K^+^ Quantification

Na^+^ and K^+^ contents of pulverized tissues were determined by nitric-perchloric acid digestion (AOAC, 1990), measured on an AA unit per Western States Version 4.00 P-4-20 (A&L Plains Analytical Laboratory, Lubbock, TX, United States). Values were presented as % Na^+^ or K^+^ per gram of sample (*n* = 3). Total Na^+^ and K^+^ were also determined for image-based phenotyping. Pulverized tissues were digested with dilute HNO_3_ (0.5M) at room temperature and the supernatant was used for flame photometry using the Model 420 Flame Photometer (Sherwood Scientific Ltd., Cambridge, United Kingdom). Na^+^ and K^+^ concentrations were calculated based on standard solution with 0.5/1 mM NaCl:KCl using the same sample dilution factor, amount, and initial sample weight as follows: [Na^+^]/[K^+^] = Reading/100 × Standard/100 × Digestion solution × Dilution factor/Dry weight × 100 (*n* = 5).

### Proline Content

Pulverized leaves (100 mg) from plants at V7 to V9 (i.e., maximum tillering) stage were homogenized in 5 ml sulfosalicylic acid (3% w/v) and centrifuged at 13,000 × *g* for 10 min to collect the supernatant. Assay solution was comprised of fresh ninhydrin (2.5% w/v), glacial acetic acid (60% v/v), and phosphoric acid (40% v/v). Supernatant (0.1 ml) was combined with 0.2 ml assay solution and 0.2 ml glacial acetic acid and was incubated at 95°C for an hour. Chromophore was extracted with 1 mL toluene, and the organic phase (0.1 ml) was recovered into 96-well microtiter plates for absorbance measurements at 520 nm (Bates et al., 1973). Quantification was based on a standard curve of 0 to 100 μM pure proline (Fisher BioReagents, BP392-100). Assays were performed with replicates (*n* = 3).

### Aggregate Phenotypic Scores (APS)

Values from each parameter were transformed to relative values on a scale of 1 to 10. The highest value was set to a score = 10, while the lowest value was set at score = 1 according: *y = 1 + (x – A) x (10 – 1)/B – A*, where, y, normalized value; x, raw value; A, minimum; and B, maximum. Scores in each genotype were summed into a normalized phenotypic score and compared to SES. Scoring matrix was analyzed by hierarchical clustering using the “pvclust” package in R (Suzuki and Shimodaira, 2006).

### Real-Time Plant Growth Profiling

The comparative panel comprised of IR29 (sensitive parent), Pokkali (tolerant parent), FL478 (tolerant RIL), FL510 (super-tolerant RIL), FL454 (sensitive RIL), and FL499 (super-sensitive RIL) were subjected to digital growth profiling under control and stress with the LemnaTec Scanalyzer 3D platform (University of Nebraska-Lincoln, Lincoln, NE, United States). Five (5) day-old seedlings grown in 0.5X Murashige-Skoog (MS) were transferred into hydroponics consisting of Turface MVP^®^ in Yoshida solution (Yoshida et al., 1971). Ten plants per genotype were loaded onto the LemnaTec Scanalyzer 3D platform after 14 days. Salinity was introduced to five plants by adding NaCl (270 mM NaCl:9.9 mM CaCl_2_) to EC = 4.5 dS m^–1^ (∼45 mM) and escalated the next day to EC = 9 dS m^–1^ (∼90 mM) for the duration of the experiment. Plants were digitally imaged daily for 18 days with RGB and hyperspectral cameras. The RGB images were analyzed using PhenoImage (Zhu et al., 2020). Plant size was initially reported as number of pixel squares, converted into cm^2^ using the Fiji software (Schindelin et al., 2012). Plant height was reported as measured by PhenoImage. Hyperspectral variances were calculated with Matlab.

### Metabolite Profiling by LC-MS/MS

Shotgun metabolite analysis was performed by liquid chromatography with tandem mass spectrometry (LC-MS/MS) at the Texas Tech University Center for Biotechnology and Genomics. Pulverized leaves (100 mg per sample) from three biological replicates (*n* = 3) of the control and stress experiments with three to five plants pooled within each RIL for each replicate were homogenized with chilled chloroform:methanol:water [1:2.5:1 (v/v/v)] and used to separate the aqueous from lipid phase. Samples were resuspended in 0.1% formic acid (v/v) and 5 μl aliquots were used for LC-ESI-MS/MS using the Dionex Ultimate 3000 nano-LC (Thermo Scientific, San Jose, CA, United States) interfaced to Q Exactive^TM^ HF Hybrid Quadrupole-Orbitrap^TM^ mass spectrometer (Thermo Scientific, San Jose, CA, United States). Metabolites were separated on a C18 Acclaim PepMap RSLC column (Thermo Scientific, San Jose, CA, United States) at constant flow of 0.3 μL/min with mobile phase solvent-A (97.9% water/2% ACN/0.1% FA) and solvent-B (99.9% ACN/0.1% FA). Metabolites were separated by following the gradient of solvent-B. For MS/MS analysis, the first scan was 50–500 m/z at mass resolution of 120,000. The ten most intense ions from the first scan were used for HCD MS/MS with elevated collision energy of 20–60% in positive and negative modes, with two replicates per pooled sample.

The Compound Discoverer v3.0 (Thermo Scientific) was used to detect compounds with “Predicted Formula,” followed by automatic online library search against the mzCloud and ChemSpider databases and validated by the mirror plot of MS/MS spectra in library standards (Pence and Williams, 2010; Mistrik, 2018). For quantitative analysis, area under peak was calculated with precursor ion mass tolerance = 5 ppm, intensity tolerance = 30%, minimum peak intensity = 1 × 10^6^, alignment mass tolerance = 5 ppm, peak alignment maximum shift = 2 min, and mass tolerance of fragment ion = 10 ppm.

### Transcriptome Profiling by RNA-Seq and Transcriptional Network Modeling

Replicated control and stress experiments were comprised of three to five individual plants for each RIL pooled within each of the three biological replicates (*n* = 3). Total RNA was extracted from frozen leaves of each replicate with the miRVana^TM^ miRNA Isolation Kit (Invitrogen, Carlsbad, CA, United States) to construct time-course (0, 24, 48, 72, and 144 h) RNA-Seq libraries. RNA-seq libraries were created from pooled samples. Strand-specific 150-bp paired-end libraries were sequenced twice on Illumina HiSeq3000. Data were analyzed using an established pipeline (Kitazumi et al., 2018). Sequence output from indexed RNA-Seq libraries were preprocessed with Cutadapt (Martin, 2011) and mapped against the Kasalath reference (Sakai et al., 2013) using HISAT2 (Kim et al., 2015). Transcript read counts were normalized by TMM (Trimmed Mean *M*-values) and differential expression was examined with edgeR with false detection rate of 0.05 (McCarthy et al., 2012).

Gene expression clusters were identified and visualized using the “MBCluster.Seq” package in R which used K-means++ clustering (Si et al., 2013). Specifically, time-course expression data were independently analyzed in each genotype to discern the co-expression clusters. Genetic networks were investigated by the mutual rank (MR) method (Obayashi and Kinoshita, 2009). Pearson’s correlation coefficient (PCC) matrix was established for each cluster at cut-off of PCC < 0.95. MR was calculated through: *MR_AB_ = Square-root of Rank_AtoB_* × *Rank*_BtoA_. Gene interactions with values <10 were excluded. Genetic networks were constructed and visualized using Cytoscape (Smoot et al., 2010). The annotation applied to RNA-Seq dataset was based on UniProt with additional expansion from relevant databases and literature (The UniProt Consortium, 2018). Metabolic pathway modeling was aided by the Kyoto Encyclopedia of Genes and Genomes (KEGG) (Kanehisa and Goto, 2000; Kanehisa et al., 2018).

### Statistical Analysis

Statistical analyses were conducted with R (R Core Team, 2020). Pearson’s correlation coefficient (PCC) matrices, gene ranks, and mutual ranks for genetic network and PCA were scripted in R-package. Tukey’s HSD *post hoc* tests following ANOVA were performed with the “agricolae” package (de Mendiburu and de Mendiburu, 2019). K-means clustering used for the metabolite analysis was done through the K-means++ code in “mytools” package (Yasumoto, 2020). The “ggplot2” package was used for data visualization (Wickham and Chang, 2008).

## Results

### Evaluation of the IR29 × Pokkali RILs Under Severe Salinity Stress

**FIGURE 1:**
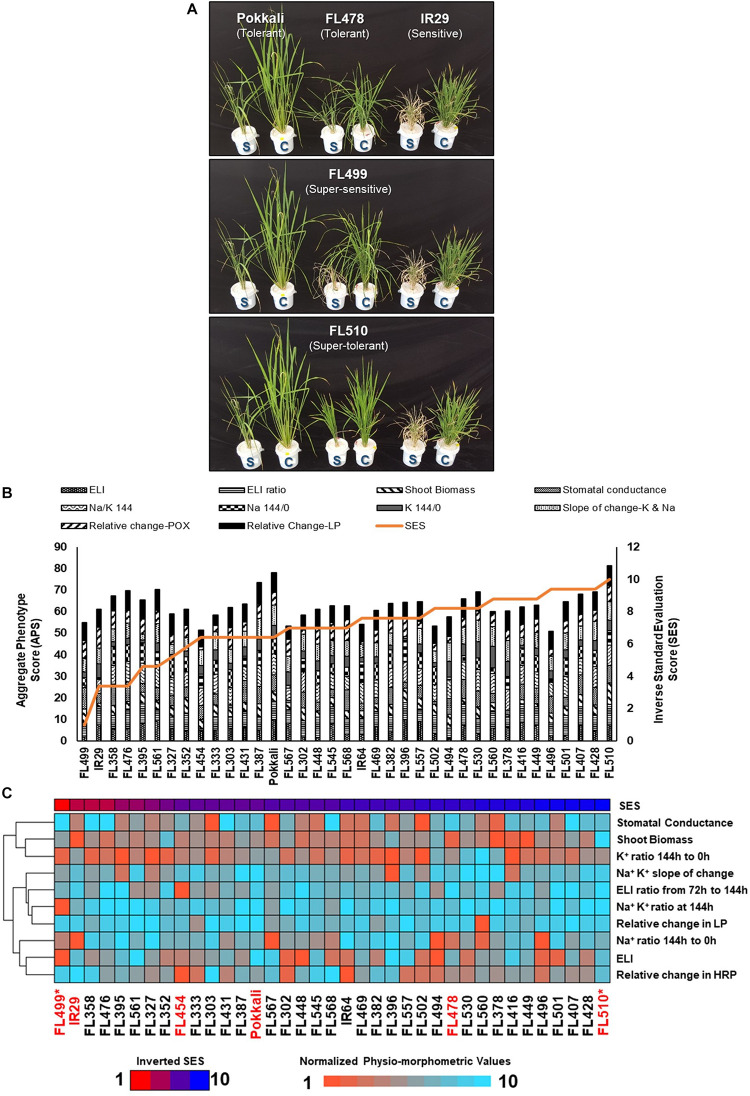
Salinity tolerance during vegetative growth window (i.e., tillering stage; V4 to V8) across the *minimal comparative panel* representing the full phenotypic range at EC = 9 dS m^–1^ (IRRI) and EC = 12 dS m^–1^ (Texas). **(A)** Comparison of plant health in control (C) and stress (S) experiments after 6 days (144 h) at EC = 12 dS m^–1^. The outliers FL510 (super-tolerant) and FL499 (super-sensitive) are highlighted compared to tolerant parent Pokkali (*Saltol* donor), sensitive parent IR29, and tolerant sibling FL478 (see Table 1). Differences in injury and growth were evident particularly between the transgressive FL510 and FL499. **(B)** Individual physio-morphometric scores were normalized and combined as *Aggregate Phenotypic Score* (APS), which assumes equal weights of each parameter that includes Electrolyte Leakage Index (ELI) and its ratio (ELI ratio) at first injury (72 h) and maximum injury (144 h), shoot biomass ratio (stress/control), stomatal conductance ratio (stress/control), Na^+^/K^+^ at maximum injury (Na/K 144/0), Na^+^ ratio at maximum injury (Na 144/0), K^+^ ratio at maximum injury (K 144/0), slope of K^+^ and Na^+^ changes (control/stress), change in peroxidase activity (change-POX; stress/control), and change in lipid peroxidation (change-LP; stress/control). APS was plotted against the inverse of SES for direct proportionality. **(C)** Heat map showing the gradients of the normalized physio-morphometric scores across the comparative panel, presented from the worst to the best SES (inverted) according to the bar graph distribution across the population in **(B)**. From this figure, traits that drag down the APS of a given genotype are more apparent. The genotypes with an asterisk (*) indicate the most transgressive lines in the population.

The IR29 × Pokkali *core mapping population* (second filial generation/F_2_ and eighth filial generation/F_8_-RILs) was initially evaluated at IRRI at early seedling growth stage (V1 to V5) under EC = 9 dS m^–1^ using shoot and root Na^+^/K^+^ and Standard Evaluation Score (SES) (Gregorio, 1997; Bonilla et al., 2002; Gregorio et al., 2002; Walia et al., 2005; Singh et al., 2007; Thomson et al., 2010). The SES ranges from 1 to 9, with 1 representing the highest tolerance. A total of 64 F_8_-RILs (*representative group*) spanning the range of tolerance across the population were selected. Along with the parents IR29 and Pokkali, and a salt-sensitive check IR64, we conducted a time-course (0, 24, 48, 72, and 144 h) evaluation of salinity stress response across the *representative groups* during an extended vegetative growth window (V4 to V12). Severe stress (EC = 12 dS m^–1^) was used to push the limits of phenotypic potentials that may not have been revealed from the earlier experiments at IRRI. We expected to identify outliers that perform worse than IR29 or better than Pokkali at V4 to V12 based on SES and results revealed clear outliers not identified at V1 to V5 (Supplementary Dataset 1). From these, we established the *minimal comparative panel*, consisting of thirty-seven RILs spanning the full range of tolerance at EC = 9 dS m^–1^ (sensitive: IR29, FL454; tolerant: Pokkali, FL478), and the outliers revealed at EC = 12 dS m^–1^, consisting of the super-sensitive FL499 and super-tolerant FL510 (Table 1 and Figure 1A).

**TABLE 1:** Summary of the Standard Evaluation Scores (SES) and qualitative phenotypic ranking at vegetative growth/tillering stage (V4 to V8) across the 67 F_8_-RILs (i.e., representative group; *n = 8*).

Genotype	SES EC = 12 (TTU)	Classification	Saltol Peak Marker (+/–)	Saltol Flanking markers (+/–)	Genotype	SES EC = 12 (TTU)	Classification	Saltol Peak Marker (+/–)	Saltol Flanking markers (+/–)
FL301	4.5		+	–	FL460	5.5		–	–
FL302	4	Moderate	+	NA	FL463	5		–	–
FL303	4.5	Sensitive	–	NA	FL468	7		–	–
FL327	5.5	Sensitive	+	+	FL469	3.5	Moderate	–	–
FL333	4.5	Sensitive	+	+	FL472	7		NA	+
FL352	5	Sensitive	–	–	FL473	4.5		+	+
FL356	7		–	–	FL476	7	Sensitive	+	NA
FL358	7	Sensitive	+	NA	FL478	3	Moderate	+	–
FL378	2.5	Tolerant	+	+	FL483	4.5		–	NA
FL382	3.5	Moderate	–	NA	FL494	3	Moderate	–	–
FL387	4.5	Moderate	+	+	FL496	2	Tolerant	+	NA
FL389	6		–	–	FL499	9	Supersensitive	NA	+
FL395	6	Sensitive	–	–	FL501	2	Tolerant	+	+
FL396	3.5	Moderate	+	NA	FL502	3	Moderate	–	–
FL397	4		–	+	FL507	7		+	+
FL399	6		–	NA	FL508	6		+	NA
FL407	2	Tolerant	+	+	FL510	1.5	Super tolerant	+	–
FL409	6		–	–	FL512	8		–	–
FL416	2.5	Tolerant	NA	+	FL530	3	Moderate	+	+
FL418	7		–	–	FL541	4		+	+
FL428	2	Tolerant	+	–	FL542	7		+	+
FL431	4.5	Sensitive	–	–	FL545	4	Moderate	+	–
FL433	4		–	–	FL557	3.5	Moderate	–	–
FL434	4.5		–	NA	FL560	2.5	Tolerant	+	+
FL435	5		–	–	FL561	6	Sensitive	+	+
FL441	7		–	+	FL567	4	Moderate	–	–
FL442	7		+	–	FL568	4	Moderate	–	NA
FL443	7		NA	–	FL572	6		–	–
FL445	7		+	+	FL577	6		+	NA
FL446	5		–	+	FL588	6		+	NA
FL448	4	Moderate	–	–	FL590	6		+	NA
FL449	2.5	Tolerant	+	–	IR29	8	Parent (Sensitive)	–	–
FL454	4.5	Sensitive	–	–	IR64	3.5/8	Check (Moderate)	NA	NA
FL456	7.5		NA	+	Pokkali	5	Parent (Moderate)	+	+

*Salt tolerance was compared with *Saltol* genotype, which was determined based on the presence of the Pokkali allele at the peak and/or flanking markers (Thomson et al., 2010).*

The *minimal comparative panel* was profiled for electrolyte leakage index (ELI), cellular ion concentration, lipid peroxidation (LP) as measure of cellular membrane injury, total peroxidase activity (POX) as measure of ROS scavenging capacity, shoot biomass, and stomatal conductance (Figure 1B). Only mild correlation with SES was established (Supplementary Table 1), including POX at the point (144 h) of maximum injury (*r*^2^ = 0.17), shoot Na^+^/K^+^ (*r*^2^ = 0.15), and POX stress/control ratio (*r*^2^ = 0.11). Collectively, the physio-morphometric parameters correlated only mildly with SES.

The tolerant FL478 with *Saltol* introgression from Pokkali was as tolerant as Pokkali under both EC = 9 dS m^–1^ and EC = 12 dS m^–1^ (Table 1 and Figure 1A; Thomson et al., 2010). Other RILs with homozygous introgression for *Saltol* had comparable SES as Pokkali and FL478, confirming the biological significance of the blind SES ranking at EC = 12 dS m^–1^ (Supplementary Dataset 1). As outlier, the super-tolerant FL510 had the best SES and highest aggregate phenotypic score (APS) across the population, significantly outperforming Pokkali and FL478. While the individual scores of FL510 were not always the best, none were below the population means, implying minute but additive effects of many factors. FL478 tended to have very good scores in some parameters but poor scores in others, causing a penalty to the net phenotypic score. Pokkali had very good APS, but its SES was inferior to FL510 (Figure 1B).

The sensitive parent IR29 along with about 30% of RILs including FL454 (sensitive) and FL499 (super-sensitive) were the worst performers based on SES alone (Supplementary Dataset 1 and Table 1). FL499 was severely injured with the worst scores for most parameters especially for ELI and Na^+^/K^+^. The trends in APS and normalized physio-morphometric values showed that the poor ranking of FL499 was due to poor scores in most parameters (Figures 1B,C). In contrast, FL510 has strong rankings in all traits observed, culminating in the best APS and contributing to its high SES. Integration of all physio-morphometric parameters into an aggregate score (APS) suggest various permutations by which different parental attributes could be combined in the progenies either optimally or non-optimally.

### Relatedness Among RILs Based on Physio-Morphometric Matrix

Based on few parameters where the sensitive parent IR29 had significantly better scores than the tolerant parent Pokkali, we hypothesized that some IR29-derived properties may be contributing to the total potential when combined with other complementary properties from Pokkali. To address this hypothesis, we used the full matrix to assess similarities across RILs by neighbor-joining dendrograms. The first measure established global relationships based on all contributing factors. The second was based only on Na^+^ exclusion components (Na^+^ content, K^+^ content, Na^+^/K^+^, and ELI) to assess the contribution of *Saltol* (Table 1).

**FIGURE 2:**
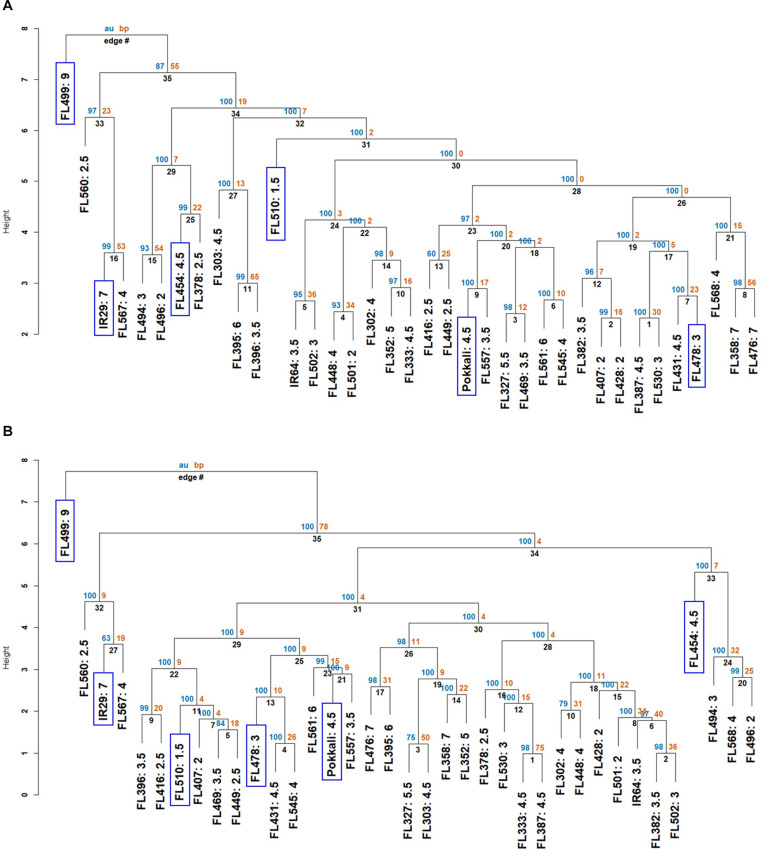
Neighbor-joining dendrograms showing two layers of similarities based on the phenotypic matrix (APS and SES). Each genotype in the dendrogram is suffixed with their SES. The individual genotypes (parents, RILs) that were investigated to understand the physiological mechanisms are highlighted in blue boxes. AU (blue) refers to the adjusted *P*-value of clustering, and BP (orange) refers to the bootstrapping value of “pvclust” package. The *y*-axis of each dendrogram refers to the degree of separation (height) between groups. **(A)** Similarities based on the entire matrix (components of APS + SES). Dendrogram shows the poor genotypes represented by IR29, FL454, and FL499 forming distinct clades from the good genotypes represented by Pokkali, FL478, and FL510. **(B)** Similarities based on APS components relevant to Na^+^ sequestration (ELI, Na^+^, and K^+^ contents at control and 144 h, Na^+^/K^+^ ratio). Dendrogram shows the tendency for individuals carrying the Pokkali *Saltol* allele to cluster together within one large clade. In both **(A,B)**, the super-sensitive FL499 had the earliest divergence.

The full-matrix dendrogram illustrates the overall range of similarities, where the super-sensitive FL499 formed the most distant clade (Figure 2A). The other poor RILs (IR29 and FL454) formed distinct clades from the better RILs (Pokkali, FL478, and FL510). The super-tolerant FL510 also formed the most distant sub-clade among the mostly good RILs, consistent with its superior tolerance. However, the large clade of mostly good RILs also included few inferior RILs, suggesting that even the poor RILs shared some attributes with their better siblings.

*Saltol* effects are primarily associated with Na^+^ exclusion. The dendrogram based on salt exclusion components revealed even more meaningful groupings (Figure 2B). The clear outliers were the inferior RILs (IR29, FL454, and FL499), all without *Saltol* (Table 1). The large clade consisting of mostly good RILs is divided into two sub-clades. The first is comprised of *Saltol-*positive RILs (tolerant Pokkali and FL478, and super-tolerant FL510), with Pokkali and FL478 being more closely related to each other than to FL510. The second is comprised of mostly (13 out of 17) good and moderate *Saltol*-positive RILs, indicating that similarities among the good RILs are largely due to *Saltol*. However, such similarities excluded the contributions of other properties in the full matrix, hence without the contributions of the genetic background.

### Superiority and Inferiority of Transgressive RILs Based on Real-Time Growth Profiling

We used the representative phenotypic classes comprised of the sensitive parent (IR29), tolerant parent (Pokkali), sensitive RIL (FL454), tolerant RIL (FL478), super-sensitive RIL (FL499), and super-tolerant RIL (FL510) to track plant growth in real-time with the LemnaTec imaging system for a period of 18 days from V4 to V12. RGB imaging determined the projected shoot area (PSA) and plant height, which correlate with plant biomass (Campbell et al., 2015). Plants were stressed at EC = 9 dS m^–1^ to remove potential bias against the sensitive classes given that EC = 12 dS m^–1^ was nearly lethal for FL499. Nevertheless, EC = 9 dS m^–1^ was strong enough to differentiate the inherent potentials across the panel.

**FIGURE 3:**
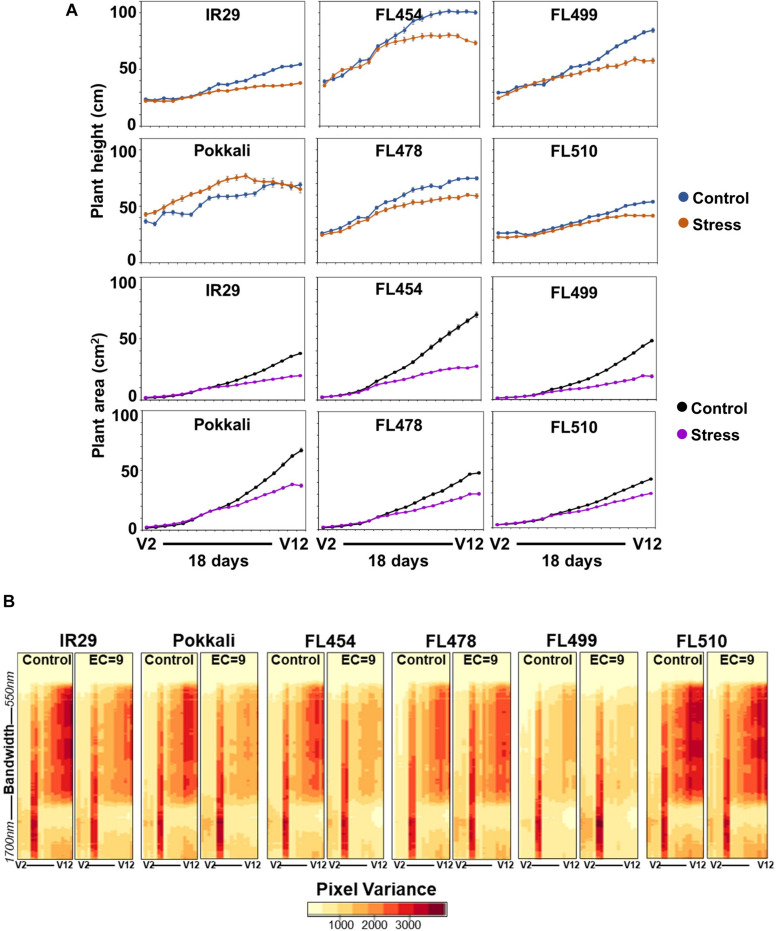
Real-time growth profiling of the sensitive parent IR29, tolerant parent Pokkali, sensitive RIL FL454, tolerant RIL FL478, super-sensitive RIL FL499, and super-tolerant RIL FL510 at EC = 9 dSm^–1^. **(A)** Growth curves as a function of changes in plant area and plant height (*y*-axis, as cm^2^ and cm, respectively) during the 18-day period of stress (*x*-axis, plotted as the vegetative growth stages V2 to V12), calculated based on pixels captured by RGB and hyperspectral camera. Note the time of forking between the control and stress curves and the angle of the fork, reflective of growth plateauing. **(B)** Variation in hyperspectral variance at 243 wavelength bands ranging from 550 to 1,700 nm. Heat maps of hyperspectral variances are potential indicators of overall plant health and stress injuries. The overall patterns in hyperspectral variances mirror the patterns revealed by the growth curve analysis.

The image-based growth curves based on daily measurements of plant area and height showed linear growth patterns over time (Figure 3A). However, the inherent growth potentials differed across genotypes even in the control. Growth rates in the control were higher in Pokkali, FL454, and FL499 relative to IR29, FL478, and FL510. Growth potentials appeared to be due to combined effects of parental attributes. For instance, the fast-growing salt-sensitive RILs (FL454 and FL499) were similar to the salt-tolerant parent Pokkali in terms of plant height, but their tillering capacity was similar to IR29. The salt-tolerant RILs (FL478 and FL510) were similar in growth pattern to the salt-sensitive parent IR29. The improved high-yielding *Xian/Indica* variety IR29 has short stature with profuse tillering, while the *Aus* landrace Pokkali has tall stature, darker green leaves, and less tillering. FL478 has the same *Xian/Indica* plant-type as IR29, while FL510 has distinct morphology (Figure 1A). It is intermediate in height, with thick, dark green, and erect leaves and stems, similar to the new plant-type (NPT) architecture of rice (Peng et al., 1994, 2008).

Growth curves indicate that stress penalty was common across all genotypes (Figure 3A). However, the magnitude of penalty varied significantly regardless of the inherent variation in growth potentials under control condition. For instance, the timing when growth retardation occurred varied with clear correlation to tolerance. Plants under control and stress grew at similar rates before stress. While growth retardation based on PSA and height was detectable as early as 7 days after stress, variation across genotypes was evident based on the timing of the forking between the control and stress curves, which determined the magnitude of growth plateauing (fork angle) in real-time (Figure 3A). Growth penalty was most severe in the sensitive IR29, FL454, and FL499 relative to their respective controls. This was also supported by tiller reduction, plant biomass, and symptoms of leaf injury. Tillering of Pokkali and FL478 were also negatively affected, but much less than IR29, FL454, and FL499. The super-tolerant FL510 had the least penalty based on tiller reduction and timing and angle of forking (Figure 3A). The PSA ratio (stress/control) also indicated difference in tolerance based on growth maintenance (Supplementary Figure 1). Tiller reduction was evident from decreasing PSA ratio in all genotypes. However, the FL510 and FL478 ratios stayed close to 1, indicating much less injury.

To further substantiate the observed variation in growth penalty, we examined the hyperspectral profiles across the growth window as indicator of plant health and vigor. Hyperspectral profiles were based on image captured at 243 wavelength bands ranging from 550 to 1,700 nm, with each band evenly separated by 4.7 nm. Image variance plots described the spread of pixel intensities as indicators of physical changes including wilting, drying, yellowing or necrosis. Differences in growth across genotypes (Figure 3A) were generally consistent with hyperspectral profiles (Figure 3B). Reduction in image variances was quite apparent in IR29 and FL454, but only minimal in FL510 and FL478, indicative of lesser injuries and more stable profiles throughout the stress period. FL499 had lower image variances even under control, suggestive of less than optimal growth that is further exacerbated by stress. Interestingly, the hyperspectral profiles of the super-tolerant FL510 in both control and stress were quite similar to the high-yielding parent IR29 under control. This suggests a positive attribute acquired by FL510 from IR29, which translated into net gains in combination with defense-related traits from Pokkali.

### Na^+^/K^+^ and Proline Profiles in Relation to Real-Time Growth Variances

The dynamics of Na^+^ and K^+^ accumulation was investigated to assess the contribution of Na^+^ exclusion capacity to growth penalty using the Na^+^/K^+^ ratio (Na^+^/K^+^) in shoots and roots as primary indicators. Variation for shoot Na^+^/K^+^ across genotypes was significant in stress but not in control (Supplementary Figure 2A). IR29, FL454, and FL499 had significantly higher Na^+^/K^+^ than Pokkali, FL478, and FL510. The mean Na^+^/K^+^ segregated the panel into tolerant (Pokkali, FL478, and FL510) and sensitive (IR29, FL454, and FL499) groups (*P* < 0.05). Because of *Saltol*, Pokkali had the lowest Na^+^/K^+^ within the tolerant group, thus the overall potentials of FL478 and FL510 may not depend on *Saltol* effects alone. Differences in root Na^+^/K^+^ were not significant (Supplementary Figure 2B).

Proline contributes to the maintenance of cellular water potential by osmotic adjustment (Ashraf and Foolad, 2007; Szabados and Savoure, 2010). The total proline content of each genotype was measured after 7 days of stress, which also corresponded to the initiation of forking between the control and stress growth curves (Figure 3A). Proline content increased significantly (*P* < 0.05) under stress in IR29 and FL454 but not in other genotypes (Supplementary Figure 2C), suggesting that osmotic adjustment is not a major factor.

### Metabolite Signatures Reflect the Coupling and Uncoupling of Parental Attributes

**FIGURE 4:**
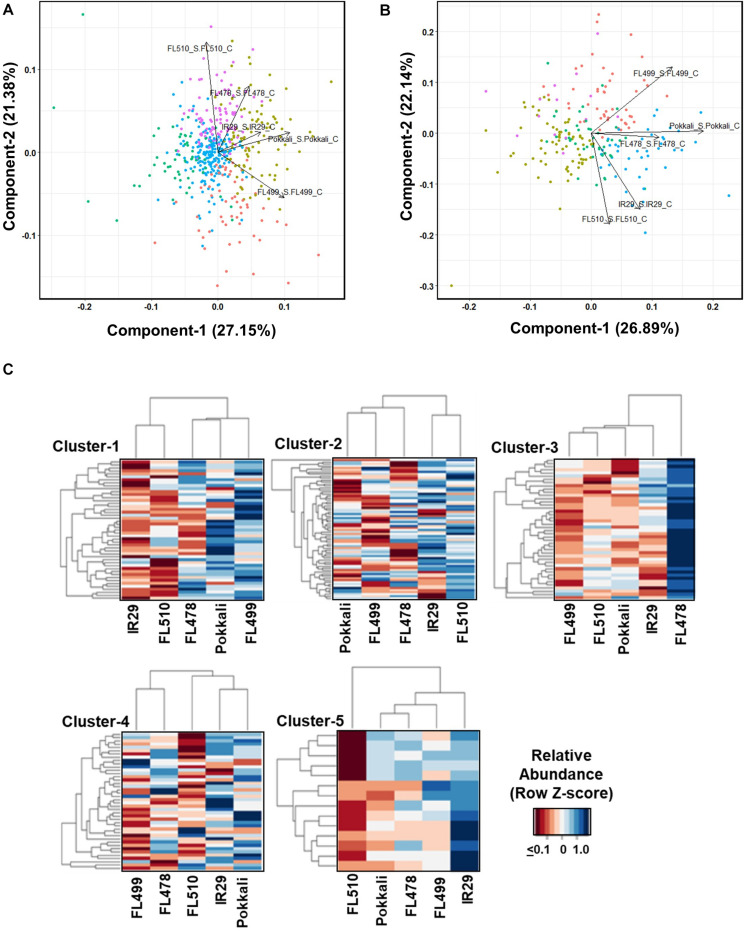
Shotgun LC-MS/MS metabolite profiles across the representative phenotypic classes at the point of maximum stress (7 days) at EC = 12 dS m^–1^. **(A)** Principal component analysis (PCA) of metabolites with common occurrence regardless between control and stress in sensitive parent IR29, tolerant parent Pokkali, sensitive FL454, tolerant FL478, super-sensitive FL499, and super-tolerant FL510. PC-1 and PC-2 separated the good RILs FL478 and FL510 from the poorest RIL FL499, accounting for 27.15% and 21.38% of phenotypic variance, respectively. The PCA also shows high similarities between the two parents. **(B)** PCA of metabolites with differential abundances between control and stress at *p* < 0.05. Based on PC-1 and PC-2, which accounted for 26.89% and 22.14% of phenotypic variance, respectively, the super-sensitive FL499 was quite distant from the super-tolerant FL510, despite the high level of similarities between the parents, hence non-additive and transgressive. The tolerant FL478 was more similar to Pokkali than to IR29, and any of its siblings. **(C)** K-means clustering heat maps and dendrograms showing the patterns of metabolite co-abundances across genotypes. Heatmap highlights a cluster that is highly abundant in each genotype. Clustering by genotype shows the similarity of IR29 and FL510, especially in clusters 1, 2, and 4. Cluster 5, highlights the differences that are contributory toward contrasting phenotypes.

Shotgun profiling by LC-MS/MS identified about 7,000 unique macromolecules, many of which were unique to a given genotype. A total of 217 known metabolites had differential abundances between stress and control at *P* < 0.05 (Supplementary Dataset 2). K-means clustering and principal component analysis (PCA) revealed multiple components that spread the entire dataset widely based on variances. FL499 had the most distinct profile (Figure 4A). The PCA plot revealed significant overlaps among the tolerant genotypes (FL478 and FL510), with patterns completely opposite from the parents (IR29 and Pokkali) and super-sensitive FL499. These trends indicate metabolite signatures associated with salinity tolerance. The super-tolerant FL510 was similar to the sensitive parent IR29, while the tolerant FL478 was similar to the tolerant parent Pokkali (Figure 4B). All these suggested that the improved high-yielding but salt-sensitive parent IR29 has significant contributions to the overall transgressive potential of FL510.

The K-means clustering revealed different patterns of co-expression (Figure 4C). Clusters-1, 2, and 4, enriched with different types of flavonoids and other antioxidants have similar profiles in IR29 and FL510 but not in other genotypes. Cluster-1 is highlighted by known indicators of oxidative stress such as (-)-allantoin and distinguishes the super-sensitive FL499 from the others (Kand’ár and Žáková, 2008). Cluster-3 distinguished the tolerant FL478 from the rest, enriched with JA-associated metabolites and fatty acids.

ABA and other related metabolites are the dominant components of cluster-4. This cluster is most similar between the tolerant parent Pokkali, sensitive parent IR29 and super-tolerant FL510, and separated the tolerant FL478 and super-sensitive FL499 from the group. Cluster-5 features the compatible osmolyte trehalose and other compounds with similar properties and distinguished the sensitive parent IR29. Overall, metabolite profiles revealed meaningful enrichments that distinguished the transgressive segregants. PCA indicates that the salt-sensitive parent IR29 has important contributions to the overall potential of the super-tolerant FL510, consistent with the trends revealed from the real-time growth profiling.

### Network Rewiring in Transgressive Segregants Based on Transcriptome Profiles

**FIGURE 5:**
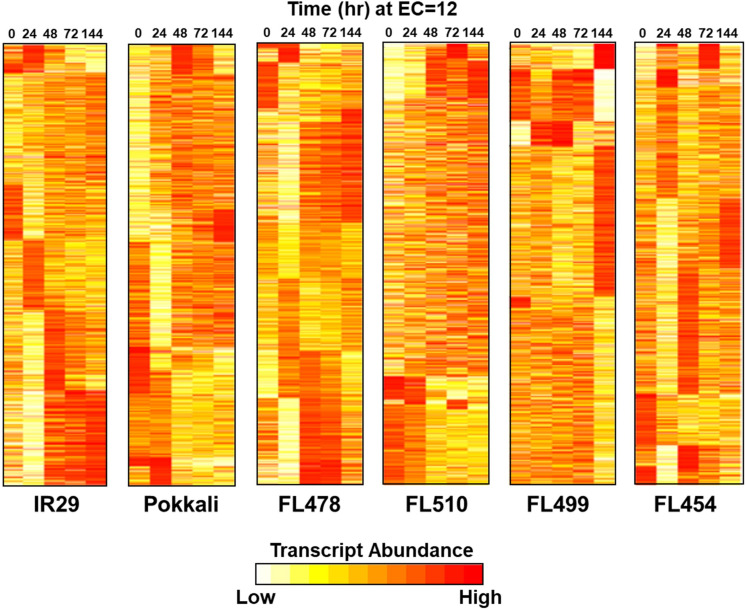
Temporal profiles of salinity stress (EC = 12 dS m^–1^) transcriptomes in parents (IR29 = sensitive; Pokkali = tolerant) and their RILs (FL454 = sensitive; FL499 = super-sensitive; FL478 = tolerant; FL510 = super-tolerant). Parallel comparison of transcriptomes across genotype shown as K-means++ clusters included 14,696 unique transcript loci. The super-tolerant FL510 is the most unique, having gradual changes in expression. The other genotypes have large clusters of co-expressed genes with drastic upregulation or downregulation across time.

To further substantiate the properties of the outliers, time-course transcriptome profiles (0, 24, 48, 72, and 144 h) were established from 44,199 transcript variants at 14,696 expressed loci (Supplementary Table 2). Contrasting profiles were evident between IR29 and Pokkali, while similarities and differences were evident across RILs (Figure 5). The super-tolerant FL510 had the highest number of genes that did not change in expression at EC = 12 dS m^–1^. Upregulation in FL510 occurred gradually but progressively compared to others. In the super-sensitive FL499, only subtle changes occurred during the early stages of stress, but many genes were drastically upregulated or downregulated at 144 h/6-day, coinciding with severe leaf senescence and necrosis. The tolerant FL478 and sensitive FL454 had large numbers of early, late, sustained, and transiently upregulated and downregulated genes similar to IR29 and Pokkali.

Genes with important roles in integrating stress and growth responses had highly significant changes in expression, including calmodulin-like *OsCML27* (Os03g0331700) that functions as a sensor for Ca^2+^-mediated stress signaling (Perochon et al., 2011), high-affinity K^+^ transporter *OsHKT7* (Os04g0607600), which is a strongly selective transporter of Na^+^ against K^+^ (Suzuki et al., 2016; Oda et al., 2018), metallothionein *OsMTI4A* (Os12g0570700) involved in oxidative defenses (Zhou et al., 2006; Yang et al., 2009; Kumar et al., 2012), and the Multi-Pass MYB-transcription factor *OsMPS* (Os02g0618400) involved in growth regulation (Schmidt et al., 2013). These genes were used to bait for other co-expressed genes (network cohorts) to detect unique signatures among the outliers as evidence of their novelty due to rewired networks.

**FIGURE 6:**
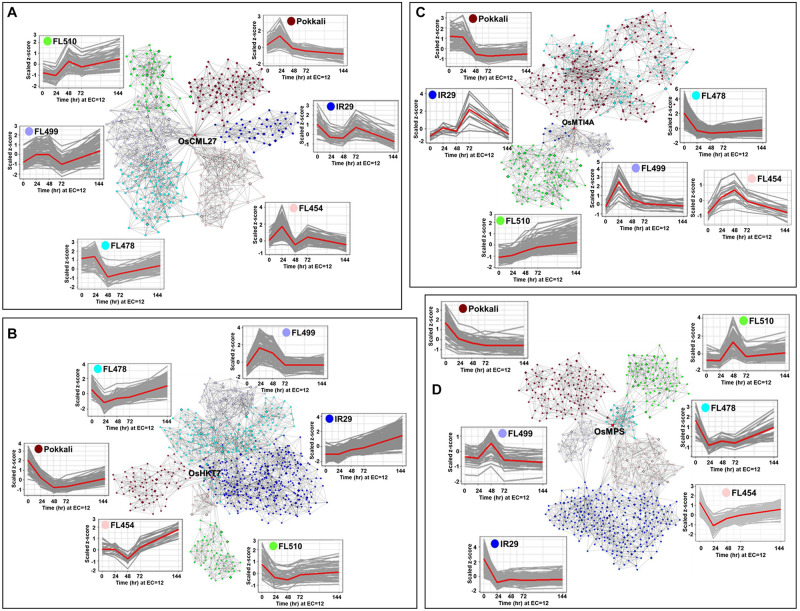
Comparison of transcriptional co-expression networks across parents and their RILs (FL454, sensitive; FL499, super-sensitive; FL478, tolerant; FL510, super-tolerant). Models represent the networks for: **(A)** *OsCML27*, **(B)** *OsHKT7*, **(C)** *OsMTI4A*, and **(D)** *OsMPS*, with important roles in developmental and stress-related responses. The temporal co-expression plots of the bait or core genes (red line) with their cohort genes (gray lines) are given for each network. Connectivity of cohort genes was based on Pearson Correlation Coefficients by mutual rank. Cohort genes are represented by nodes and co-expression is reflected on the edges.

The *OsCML27* profile was most distinct in the super-tolerant FL510 with sustained upregulation. The parents IR29 and Pokkali did not share any cohort genes with any progeny, while only minimal overlaps at best occurred between FL510, FL478, FL499, and FL454 (Figure 6A). These trends illustrate the non-additivity (network rewiring) of parental gene expression in the progenies, where outlier trends were evident. The *OsCML27* cohorts in FL510 were enriched with salicylic acid (SA) and jasmonic acid (JA) associated genes with sustained upregulation through 144 h (Supplementary Dataset 3). In contrast, distinct subsets of transiently upregulated genes occurred in other RILs, enriched with functions associated with signal transduction, cell wall biogenesis, and growth. Transient expression among the poor RILs suggests a potential interruption of growth. In the other poor RILs, the *OsCML27* cohorts also included downregulated genes associated with stress adaptation such as *OsFBK12* (Os03t0171600-02; Delayed senescence Kelch-repeat F-box protein), *OSISAP1* (Os09t0486500-02; Cold, drought and salt tolerance-associated A20/AN1 zinc-finger), *OsPLC4* (Os05t0127200-02; Salt and dehydration regulated Phospholipase C4), *OsNAGK1* (Os04t0550500-05; Drought-regulated N-acetylglutamate kinase-1), *OsMST6* (Os07t0559700-00; Monosaccharide transporter involved in water retention and membrane stability), and *OsHrd3* (Os03t0259300-01; Unfolded protein and apoptosis response; Supplementary Dataset 3) (Mukhopadhyay et al., 2004; Chen et al., 2013; Ohta and Takaiwa, 2015; Deng et al., 2019; Liu et al., 2019a). The superiority of the FL510 *OsCML27* network is further illustrated by the distinct upregulation of *OsIAA1* (Os01t0178500-02; auxin repressor AUX/IAA1), suggesting the interruption of auxin-mediated growth signaling in the poor RILs (Song et al., 2009).

*OsHKT7* was upregulated in IR29 but downregulated in Pokkali (Figure 6B). The super-tolerant FL510 resembled the Pokkali profile, while the tolerant FL478 and sensitive FL454 were similar to IR29, and the super-sensitive FL499 had non-parental profile. The *OsHKT7* cohorts had the most overlap between FL510 and Pokkali, enriched with functions associated with transcription, translation, and cell division (Supplementary Dataset 4). However, the cohorts in FL510 were uniquely enriched with cation transport functions including *OsMTP9* (Os01t0130000-01) for manganese transport, and *OsCDT2* (Os06t0143100-01) for cadmium exclusion (Kuramata et al., 2008; Ueno et al., 2015). The cohorts in IR29, FL454, FL499, and FL478 were associated with stress sensitivity highlighted by *OsMYB30* (Os02t0624300-01) and *OsHSFB2B* (Os08t0546800-01) (Xiang et al., 2013; Lv et al., 2017).

The metallothionein-encoding *OsMTI4A* was downregulated in Pokkali and transiently upregulated in IR29 (Figure 6C). Expression in FL478 was similar to Pokkali, while expression in FL499 and FL454 were similar to IR29. Most interestingly, expression in FL510 was distinct. While significant overlaps were evident between FL478 and Pokkali, and between IR29, FL454, and FL499, the cohort genes in FL510 were also distinct from the rest (Supplementary Dataset 5). Networks in Pokkali and FL478 included downregulated genes involved in translation, cell division and growth regulation, such as Cyclin F2-2 (Os02t0605000-01) and Cyclin B1-5 (Os05t0493500-00) (La et al., 2006). Networks in the poor genotypes IR29, FL454, and FL499 were comprised of transiently upregulated genes involved in general stress response. The unique *OsMTI4A* network in super-tolerant FL510 was characterized by sustained co-upregulation of genes involved in hormonal, growth and stress signaling such as *BZR3* (Os06t0552300-01; repressor of brassinosteroid signaling), *OsIAA13* (Os03t0742900-01; Auxin repressor AUX/IAA13), *CDKG2* (Os04t0488000-02; cyclin-dependent kinase G-2), *OsE2F1* (Os02t0537500-01; Mitotic cycle E2F protein), and extensin (Os01t0644600-00) (Kosugi and Ohashi, 2002; Tank and Thaker, 2011; Kitomi et al., 2012; Schmitz et al., 2013; Draeger et al., 2015). *OsMTI4A* network in FL510 suggests its unique ability to integrate stress response and growth.

The MYB-type Multi-pass transcription factor *OsMPS* is a critical regulator of plant growth and development under stress (Schmidt et al., 2013). While this gene was significantly downregulated in both parents, different patterns of transgressive upregulation were evident across the RILs (Figure 6D). Cohorts in FL510 is enriched for JA and SA signaling genes such as *OsWRKY3* (Os03t0758000-01), *OsWRKY67* (Os05t0183100-01), *OsWRKY53* (Os09t0334500-01), *OsWRKY74* (Os05t0343400-01), *MYC2* (Os10t0575000-01) and *RERJ1* (Os04t0301500-01) (Supplementary Dataset 6). These genes are important for integrating stress with development and shared at least partially with FL478 (Ramamoorthy et al., 2008; Miyamoto et al., 2013; Ogawa et al., 2017).

The *OsMPS* network in Pokkali was enriched with genes associated with general transcription and translation, reinforcing the trends observed in its other networks. The other genotypes had few similarities with *OsCML27*, *OsHKT7*, and *OsMTI4A* networks with the downregulated oxidative stress genes such as *Prx19* (Os01t0787000-01; peroxidase), *Prx117* (Os08t0113000-01; peroxidase), and *OsSIK1/OsER2* (Os06t0130100-01; LRR receptor-like serine/threonine kinase), and other genes involved in cell wall biogenesis (Cosio and Dunand, 2008; Ouyang et al., 2010). These trends are consistent with the relatively high growth penalties as shown by real-time growth profiling.

Despite the similar *OsMPS* expression in the super-sensitive FL499 and super-tolerant FL510, much of their cohort genes were distinct. Targets of *OsMPS* that were co-upregulated in FL510 were not upregulated in FL499. This suggests a possible complete uncoupling of the upstream regulator and its target effectors in FL499, and such uncoupling may only be partial in the other genotypes. Overall, the transcriptional networks support the inherent uniqueness of FL510 based on the combined effects of parentally derived and rewired networks.

**FIGURE 7:**
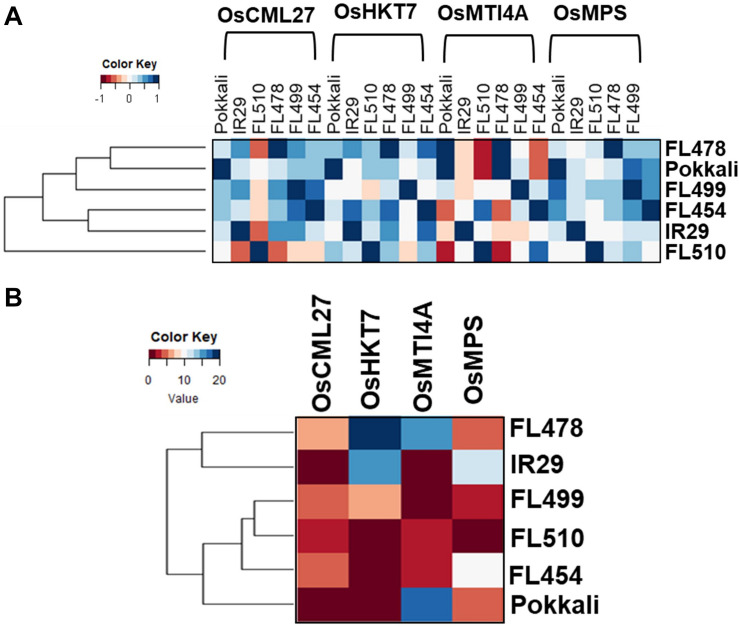
Relatedness of the genotypes according to the gene networks in Figure 6. Similarities among the genotypes were assessed by coefficient of correlation among median expression values of network components **(A)**, and by the number of shared cohort genes **(B)**.

To further illustrate the transgressive nature of FL510 and FL499, the correlation coefficients of median expression values of cohort genes were used as measure of similarity across the genotypes (Figure 7A). FL510 had the lowest correlation with all other genotypes. Similarly, the transgressive nature of the super-tolerant FL510 and super-sensitive FL499 was supported by the correlation coefficients based on the number of cohort genes that overlap across the genotypes, where FL510 and FL499 clustered together (Figure 7B). On the other hand, IR29 and FL478 shared many more genes amongst them than with the other genotypes. FL478 was selected for *Saltol* and for its overall similarity with IR29.

### Modulation of Metabolic Rates as Positive Effect of Network Rewiring

Adjustment of growth potential is the core of the uniquely superior attributes of FL510. Such potential appeared to be due to the coupling of good properties from either parent or uncoupling of bad properties from the good properties from the same parent. To address this hypothesis, we examined the similarities in primary metabolic status by reconstructing models of transcriptional states for glycolysis and tricarboxylic acid (TCA) cycles, starch metabolism, and nitrogen assimilation and transport pathways to gauge the differences in the state of plant growth across genotypes.

**FIGURE 8:**
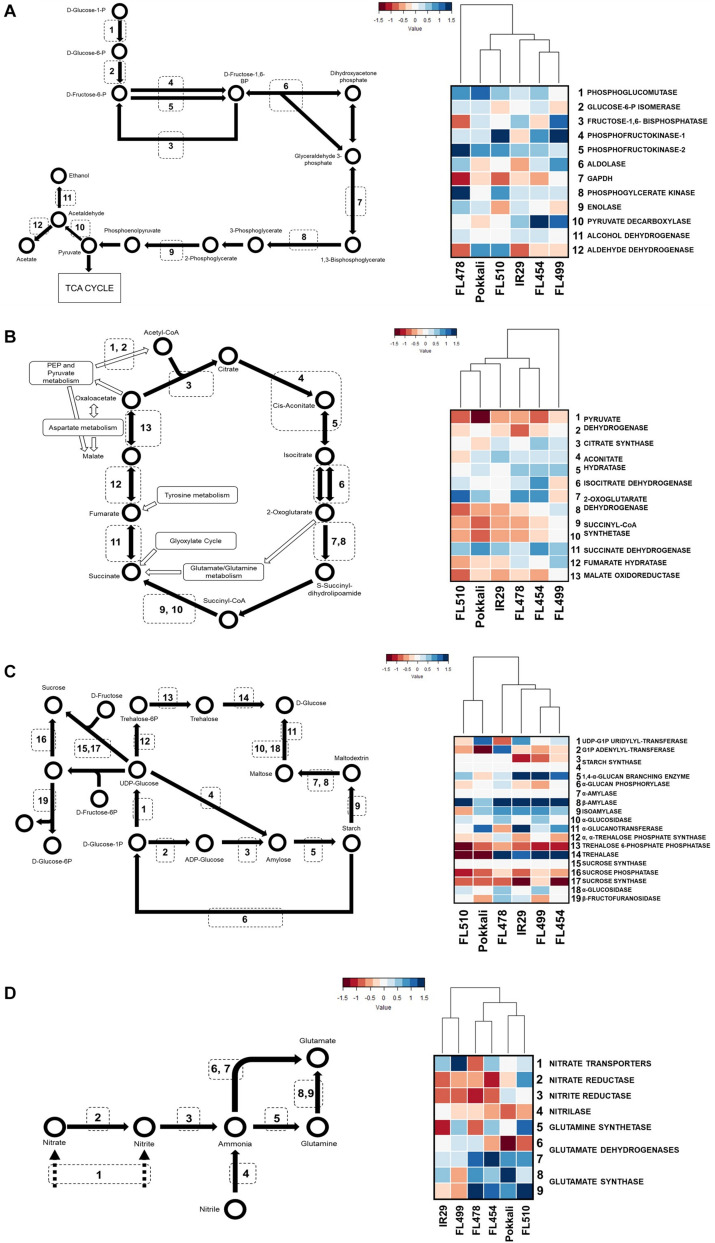
Metabolic similarities based on transcriptome profiles. KEGG models of metabolic pathways are shown on the left panel for glycolysis **(A)**, TCA cycle **(B)**, starch/sucrose metabolism **(C)**, and nitrogen assimilation **(D)**. Transcripts for enzymes in each step were mapped against each pathway. Pathway activities and the pattern of similarities across genotypes are depicted by hierarchical clustering dendrograms based on maximum log_2_-fold change in transcript abundances at the time-point with the widest difference from control. FL510 and Pokkali share similarities in repression of the carbon metabolism pathways, while nitrogen assimilation is induced in both genotypes. In comparison, the poor genotypes cluster together.

In general, glycolysis, TCA cycle, and starch and sugar metabolism had lower expression in the tolerant parent Pokkali and super-tolerant FL510 compared to the inferior genotypes (IR29 and super-sensitive FL499) where upregulation was evident (Figures 8A–C). These trends suggest that metabolic rates are slightly faster in poor genotypes, perhaps as consequence of the need to rapidly replenish metabolic intermediates as they are depleted by cellular adjustments and defense. Superior genotypes appeared to maintain better balance and less perturbation. Nitrogen metabolic and transport pathways were more strongly upregulated in superior genotypes (Figure 8D). As nitrogen assimilation is critical for vegetative growth, these trends are consistent with growth maintenance even under severe stress, consistent with the results of real-time growth profiling. Overall, in terms of the primary metabolic profiles, the super-tolerant FL510 was more similar to the tolerant parent Pokkali except for Calvin cycle (Supplementary Figure 3). Modulation of metabolic pace in FL510 may be advantageous under perturbed physiological state to minimize penalty to growth and development.

## Discussion

Transgressive segregation in natural populations could nucleate adaptive speciation (Rieseberg et al., 1999; Wagner and Lynch, 2010). The genetic basis has been attributed to complementation and additive effects, and positive or negative epistatic interactions (Dittrich-Reed and Fitzpatrick, 2013). In plant breeding, where hybridization of individuals with wide phenotypic contrasts serve as the foundation for stacking desired traits, transgressive segregation could be a common occurrence. However, novelties are often undetected due to small population size, narrow scope and limited resolution of phenotyping, limited generation time to break genetic linkage and physiological drags, and lack of sufficient data and models for prediction. Nevertheless, when interfaced with high-resolution genomic modeling, transgressive segregation could provide a fine-tuned combination of compatible and additive traits beyond what could be achieved by functional genomics alone (de los Reyes, 2019).

In marker-assisted breeding, individuals carrying few complementary major-effect QTL are crossed to maximize genetic gains through the combining potentials of each donor in the absence of linkage drags (Collard and Mackill, 2008). While much success has been achieved using this paradigm for adaptive traits and yield potential, QTL stacking tends to have the simplistic expectations that most outcomes are positive additive effects, underplaying the potential negative interactions that pollute the full force of positive synergies. Negative interactions are in fact coming from the background (minor or peripheral components) that cannot be explained by the major-effect QTL alone (Omnigenic Theory).

By systems-level approach to phenotyping, this study intended to illuminate the various types of synergies that create either large positive or negative net gains to complement what has been revealed by QTL mapping. We addressed the physiological coupling-uncoupling and network rewiring hypotheses by revealing that the inferior parent IR29 was the source of positive growth and developmental attributes that were complemented by the defense attributes of superior parent Pokkali. Additive effects are manifested in the absence of physiological drags that undermine the full expression of growth and developmental attributes and defense attributes either independently or interactively. To understand the subtleties of physiological coupling-uncoupling and network rewiring, we borrowed the paradigm of “*personalized genomics medicine*,” which considers any two individuals with similar phenotypes to have unique signatures based on multi-dimensional criteria. We scrutinized the extended phenotypic range at the highest resolution of phenotypic profiling possible. This study represents an important advance to further explore transgressive segregation for novel adaptive traits that may not be achieved through transgenics or gene editing alone.

### Evidence of Coupling-Uncoupling Effects at Multiple Levels

**FIGURE 9:**
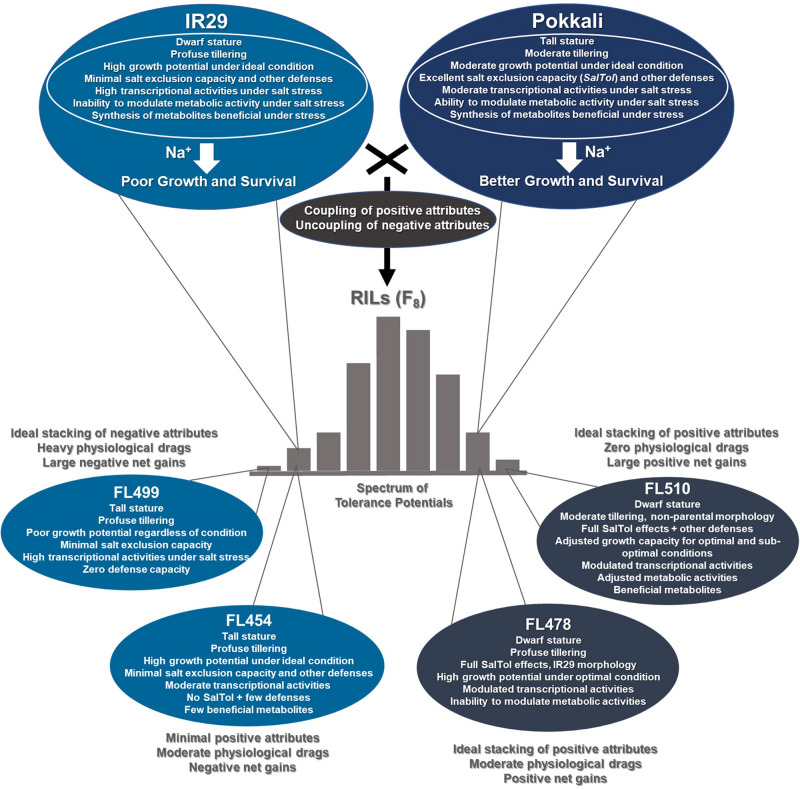
Hypothetical model of physiological coupling and uncoupling in transgressive segregants for salinity tolerance across the IR29 × Pokkali recombinant inbred population based on macro-physiological, biochemical, and molecular profiles according to de los Reyes (2019). This model proposes that the novelties of FL510 and FL499 are due to the coupling in the progeny of the good properties coming from either parent or uncoupling of bad properties from the good properties from the same parent. On top of the core mechanisms that contribute to a large proportion of phenotypic variance for defense potentials, each parent has their own characteristics that may or may not be beneficial under stress. Benefit from IR29 would be its superior growth and development potentials. Pokkali offers many stress defense mechanisms including salt exclusion. Combining the physiological potentials of parents with the reconfigured (non-parental) properties led to positive or negative coupling and uncoupling effects in RILs.

The transgressive properties of super-tolerant FL510 stem mainly from complementation of beneficial traits (coupling) and shedding of potential drags (uncoupling) from both parents, created by fortuitous genome shuffling across eight generations (F_8_). Coupling and uncoupling effects were evident at the macro-physiological, metabolome, and transcriptome levels, illustrating how the assemblages of compatible properties or their opposites configured unique attributes (Figure 9). A robust measure of variance for salinity tolerance was the magnitude of growth penalty. Sub-optimal conditions slow down photosynthesis, while requiring more resources to feed into the maintenance of metabolic stability and short-term defenses (Munns and Gilliham, 2015). High-resolution phenotyping indicated similarity in the stress response capacity of FL510 and Pokkali, but clear differences in growth properties. These genotypes had the highest aggregate phenotypic scores (APS), indicating that FL510 inherited stress-related traits from Pokkali, most prominently salt-exclusion capacity through *Saltol* (Supplementary Figures 1A,B). Evidently, the tolerant genotypes (Pokkali, FL510, and FL478) clustered together based on salt exclusion. FL478 has *Saltol*, yet it was outperformed by FL510, indicating that factors beyond salt-exclusion alone optimize the expression of tolerance.

There appears to be a threshold as to how much salt-exclusion alone can effectively provide protection if other additive and non-antagonistic properties are not in place. Prolonged stress will eventually damage the plant regardless of its inherent defense potential. One example related to the damage incurred eventually relates to the reduction of transpiration, as water and nutrient uptake, together with gas exchange, still needs to happen for maintenance. Thus, Na^+^ is still inevitably transported into the plant along with its toxic effects. Growth profiling indicated that the growth and developmental attributes inherited by FL510 from the salt-sensitive IR29 are positively complementing other attributes from the salt-tolerant Pokkali (synergism). For instance, smaller stature and low tillering habit distinguished FL510 and FL478 from their inferior siblings, and these traits offered fitness benefits as they are much easier to support energetically when metabolic intermediates are constantly being diverted toward cellular adjustments and defenses. In contrast, the inferior FL454 and FL499 exhibited less capacity for growth modulation, as indicated by tall stature (from Pokkali), profuse tillering habit (from IR29) or both. Survival is prioritized over continued growth hence the growth curve plateaus prematurely. Higher PSA ratio was observed in IR29, Pokkali, and FL478 during the onset of stress, reflecting an attempt to increase water uptake and turgor pressure for short-term stress mitigation.

The deviation (forking) of growth curves is a measure of how much metabolism has been hampered, and the penalty due to injuries and perturbed cellular processes. Being the major trade-off of survival, this deviation was minimal in tolerant genotypes especially FL510. In this context, coupling of morpho-developmental features from IR29 with the stress-response mechanisms from Pokkali is ideal, allowing the plant to maximize its survival, while minimizing trade-offs by reaching the peak of vegetative growth earlier with minimal costs. The super-sensitive FL499 had to cope with stress through an inferior mechanism from IR29, while trying to maintain its inherent high-growth, leading to a drastic growth decline and extreme salt-sensitivity. Metabolite profiles supported the complementary factors of IR29 toward a transgressive phenotype. Metabolites were similarly expressed between FL510 and IR29, indicating beneficial factors from IR29. Meanwhile, cluster-5 indicated the “*drags*” that have been uncoupled, hence increased positive net gain in FL510. Cluster-4 could also represent some of the “*drags*” that prevented FL478 from achieving a full potential comparable to FL510.

The robust transcriptome in FL510 represents an optimal mechanism characterized by progressive upregulation or downregulation across time. Responses are either for long-term adaptation or short-term adjustment. In the other inferior genotypes, gene expression was less orderly, indicative of perturbed state rather than adaptation. The transcriptional networks for four genes (*OsCML27, OsHKT7, OsMTI4A*, and *OsMPS*) with important roles in development and stress highlighted the unique configuration of FL510 as the most deviant from parental profiles (rewired networks). FL510 also inherited the repressed carbon metabolism and enhanced nitrogen metabolism of Pokkali. All these factors created synergies for robust growth through efficient integration of developmental and stress responses (de los Reyes et al., 2018). In contrast, the inferior siblings have either partial beneficial characters (FL478), stacking of detrimental characters and sensitivity properties of IR29 (FL454), or stacking of physiological drags that exacerbate the baseline sensitivity from IR29 (FL499). In the case of FL499, profuse growth was unsustainable and aggravated by inability to mitigate the toxic effects of Na^+^.

Therefore, the positive net gain in super-tolerant FL510 and negative net gain in super-sensitive FL499 can be defined by multiple coupling-uncoupling of physiological mechanisms (Figure 9). While FL478 had similar traits as FL510, it is slightly polluted with residual physiological drags that undermine its full potential. FL478 also had the morpho-developmental attributes of IR29. Evidently, sensitive genotypes are compromised by unsustainable metabolic requirement due to active growth, while lacking effective stress response mechanisms. At the transcriptome level, ideal synergism in FL510 allows for sustained response. In contrast, inferior genotypes must respond immediately, leading to an overreaction, which is energetically wasteful and detrimental in the long-term.

### Relevance of Coupling-Uncoupling Effects to the Omnigenic Theory

The Omnigenic Theory represents a modern view for explaining the genetic causes of complex traits beyond polygenic effects (Boyle et al., 2017). Complex traits are controlled by many genes/loci scattered throughout the genome, rather than a small group detected by QTL mapping, genome-wide association, or reverse genetics. The peripheral genes/loci are below the threshold of detection by QTL or reverse genetic analysis, as they have immeasurable effects as minor network components. However, their cumulative effects could equal or surpass the core effects (Liu et al., 2019b). To maximize expression potential, combinations of core and peripheral alleles and their interactions must be optimal.

Transgressive segregants are consequences of the complementation of compatible traits from both parents and absence of physiological or linkage drags. However, uncoupling events could also result in the loss of positive synergies (de los Reyes, 2019). This study illustrated that complementation in either synergistic or antagonistic fashion creates transgressive properties in both ends of the spectrum, extending beyond the typical salt exclusion mechanism from Pokkali. In FL510, growth reduction was minimized since metabolic impairment was not as extensive. FL478 and Pokkali both possess salt exclusion mechanisms, but they lack many complementary but minute effects for incremental enhancements. Salt exclusion mechanism reduces Na^+^ toxicity in metabolically active tissues, which works together with lower metabolic requirement for growth and development. Transport of Na^+^ and Cl^–^ alone constitutes major cellular energy costs. When minimized, it allows the plant to survive longer under saline environment (Tyerman et al., 2019; Munns et al., 2020). The collective responses uncovered in FL510 are consistent with the Omnigenic Theory, as no single mechanism is truly adequate to explain its extreme phenotype. The same thing is true for the super-sensitive FL499 with antagonistic effects. Physiological gain is the result of ideal synergy, supporting that the majority of the genome contributes to full physiological potential.

### Comprehensive Phenotyping and Genomic Modeling of Transgressive Traits

The transgressive salt-tolerance of FL510 may not extend to other types of stress. Indeed, other sibling RILs have been observed to survive under severe water-deficit better than FL510 (Gendron, 2019). While cases of transgressive properties such as in FL510 and FL499 may be rare in a population, individuals that exceed the parental range may be more common than perceived. Repeated recombination can create incremental improvements that create slight advantages over the parents, depending on the genetic distance between the parents. Often, individuals that display such properties are cast aside, as they do not offer much advantage in a constrained plant breeding pipeline because of linkage drags. However, these individuals offer invaluable tools in modeling how transgressive physiological properties arise in a population.

The resolution of few major QTL markers may not have predicted the transgressive phenotypes observed in this study, but genomic modeling may be a more promising approach. Genomic selection promises to identify desirable but rare recombinants for complex multi-loci interactions by taking into consideration the core and peripheral components and all forms of interactions (de los Reyes, 2019). Increasing the accuracy of modeling is dependent on the number of individuals in the modeling population. With deep individual data points, a wider coverage of possible permutations of recombinants can be mined for genomic patterns that lead to transgressive phenotypes. Thus, the interaction networks from both core and peripheral components may be modeled for their individual weights. The process from which transgressive individuals arise in plant breeding may be similar to how new phylogenetic lineages develop in natural populations (Rieseberg et al., 1999; Dittrich-Reed and Fitzpatrick, 2013). All progeny in a cross is potentially useful for modeling transgressive phenotypes. Each recombination event adds a possible permutation that should not be discarded to identify rare cases of synergism.

It is also important to increase the resolution of analysis with multiple layers of data at population-scale with detailed scrutiny of critical individuals. A multi-tier view of phenotypic range will allow a comprehensive integration of “*omics*” data to unravel the wirings of transgressive phenotypes, analogous to the paradigm of *personalized genomics medicine*. This study represents a serious attempt to test this approach that is unconventional even in modern plant breeding pipelines. While it has limitations, future technology will allow a more seamless data integration. Addition of epigenomic and chromatin profiles will certainly enhance the predictive strength of modeling (de los Reyes, 2019). The value of a genotype as donor cannot be predicted from phenotype alone unless its combining potential has been examined. Systems-level phenotyping could reveal cryptic beneficial characters that are overshadowed by antagonistic traits.

## Data Availability Statement

The RNA-Seq datasets used in this study are publicly available through the NCBI Short Read Archive (PRJNA378253: SRR11528269–SRR11528295).

## Author Contributions

IP and BR designed the experiments and wrote the manuscript. IP, AK, KC, BD, MZ-M, and HW performed the experiments and analyzed the data. RS and GG generated the RIL population. BR conceptualized the whole project. All authors contributed to the article and approved the submitted version.

## Conflict of Interest

The authors declare that the research was conducted in the absence of any commercial or financial relationships that could be construed as a potential conflict of interest.

## Supplementary Material

Supplementary Figure 1 Stress to control ratios of projected shoot area (PSA) and plant height among the different genotypes through the duration of real-time growth imaging. Ratios of stress and control measurements of PSA and plant height were plotted for all the genotypes in the same time frame as Figure 1. This was used to assess the extent of growth penalty incurred by each genotype through the stress period. Ratio values greater than 1 indicate a larger value for the stress treatment compared to the control, while those lower than 1 indicate a reduction in growth relative to the control.

Supplementary Figure 2 Physiological characterization of the representative genotypes used in the real-time growth profiling at EC = 9 dS m^–1^. Physiological indicators of cellular defense and adjustment potentials during the 18-day period at EC = 9 were evaluated including Na^+^/K^+^ in the shoot **(A)**, Na^+^/K^+^ in the root **(B)**, and total proline content in leaves **(C)**. For the Na^+^/K^+^ analysis **(A,B)**, bar graphs represent the mean Na^+^/K^+^ (*n* = 5) with standard errors. One-way ANOVA with an HSD test (α = 0.05) was used to determine significant differences between genotypes and treatments. Lower-case letters signify treatment groups, separated by significant mean differences, with “*a”* representing the highest means. For the proline content analysis **(C)**, bar graphs represent the means of total proline in leaves (*n* = 3) with standard error bars. One-way ANOVA with HSD test (α = 0.05) was used to determine significant differences between the different treatments. Lower-case letters signify treatment groups separated by significant mean differences, with “*a”* representing the highest means.

Supplementary Figure 3 Pathway induction heatmaps under stress for genes in the Calvin cycle. Values were generated from the fold-change under control conditions and the stress time point with the widest margin from control. The genotypes were hierarchically clustered to show similarity in pathway induction. RuBP, Ribulose 1,5-bisphosphate; RUBISCO, Ribulose-1,5-bisphosphate carboxylase/oxygenase; 1,3-BPG, 1,3-bisphosphoglycerate; PPK, Phosphoglycerate kinase; 3-PG, 3-phosphoglycerate; GAPDH, Glyceraldehyde-3-phosphate dehydrogenase; GAP, Glyceraldehyde-3-phosphate; TPI, Triose-phosphate isomerase; DHAP, Dihydroxyacetone phosphate; G3P, Glyceraldehyde 3-phosphate.

Supplementary Table 1 *R*^2^ and *P*-values of different traits tested in the phenotyping of the IR29 × Pokkali RIL population relative to SES. Traits with significant relationship with SES based on regression analysis (*P* < 0.05) are marked with an asterisk (^∗^).

Supplementary Table 2 Summary and mapping statistics of RNA-Seq libraries constructed for the parental genotypes and selected RILs.

Supplementary Dataset 1 F_8_-RILs generated from IR29 × Pokkali and their standard evaluation scores (SES) from initial testing at IRRI and subsequent testing at TTU.

Supplementary Dataset 2 Metabolites identified in through LC-MS/MS with significant difference between stress and control conditions (*P* < 0.05).

Supplementary Dataset 3 Gene loci co-expressed with *OsCML27* in each genotype.

Supplementary Dataset 4 Gene loci co-expressed with *OsHKT7* in each genotype.

Supplementary Dataset 5 Gene loci co-expressed with *OsMTI4A* in each genotype.

Supplementary Dataset 6 Gene loci co-expressed with *OsMPS* in each genotype.
