# Supplementary figures and images for: Novel and Transgressive Salinity Tolerance in Recombinant Inbred Lines of Rice Created by Physiological Coupling-Uncoupling and Network Rewiring Effects
Source: Front Plant Sci. 2021 Feb 23;12:615277. doi: 10.3389/fpls.2021.615277 (PMC7940525; doi:10.3389/fpls.2021.615277)

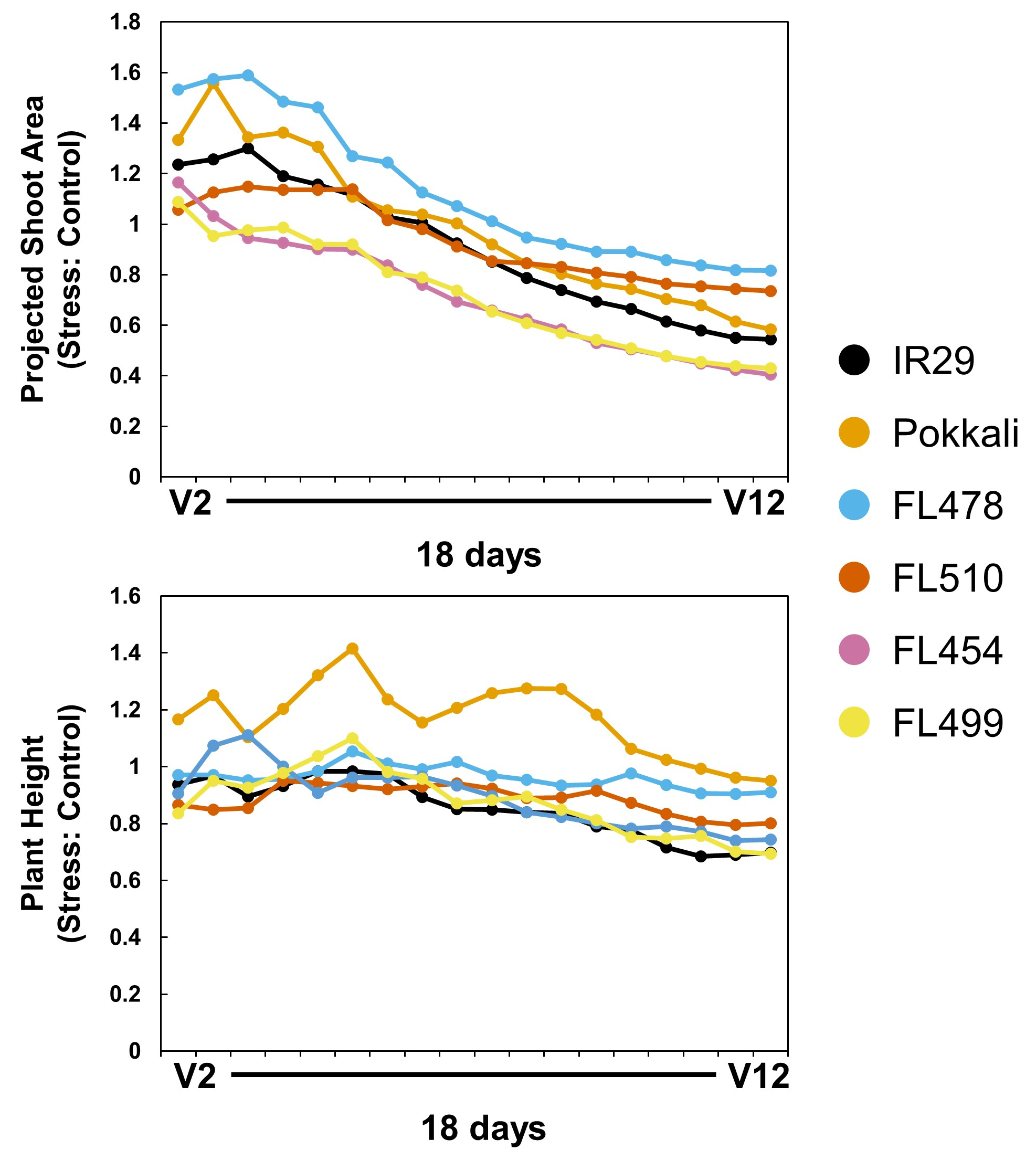

Supplement: Supplementary Figure 1 — Stress to control ratios of projected shoot area (PSA) and plant height among the different genotypes through the duration of real-time growth imaging. Ratios of stress and control measurements of PSA and plant height were plotted for all the genotypes in the same time frame as Figure 1. This was used to assess the extent of growth penalty incurred by each genotype through the stress period. Ratio values greater than 1 indicate a larger value for the stress treatment compared to the control, while those lower than 1 indicate a reduction in growth relative to the control. [file Image_1.JPEG]

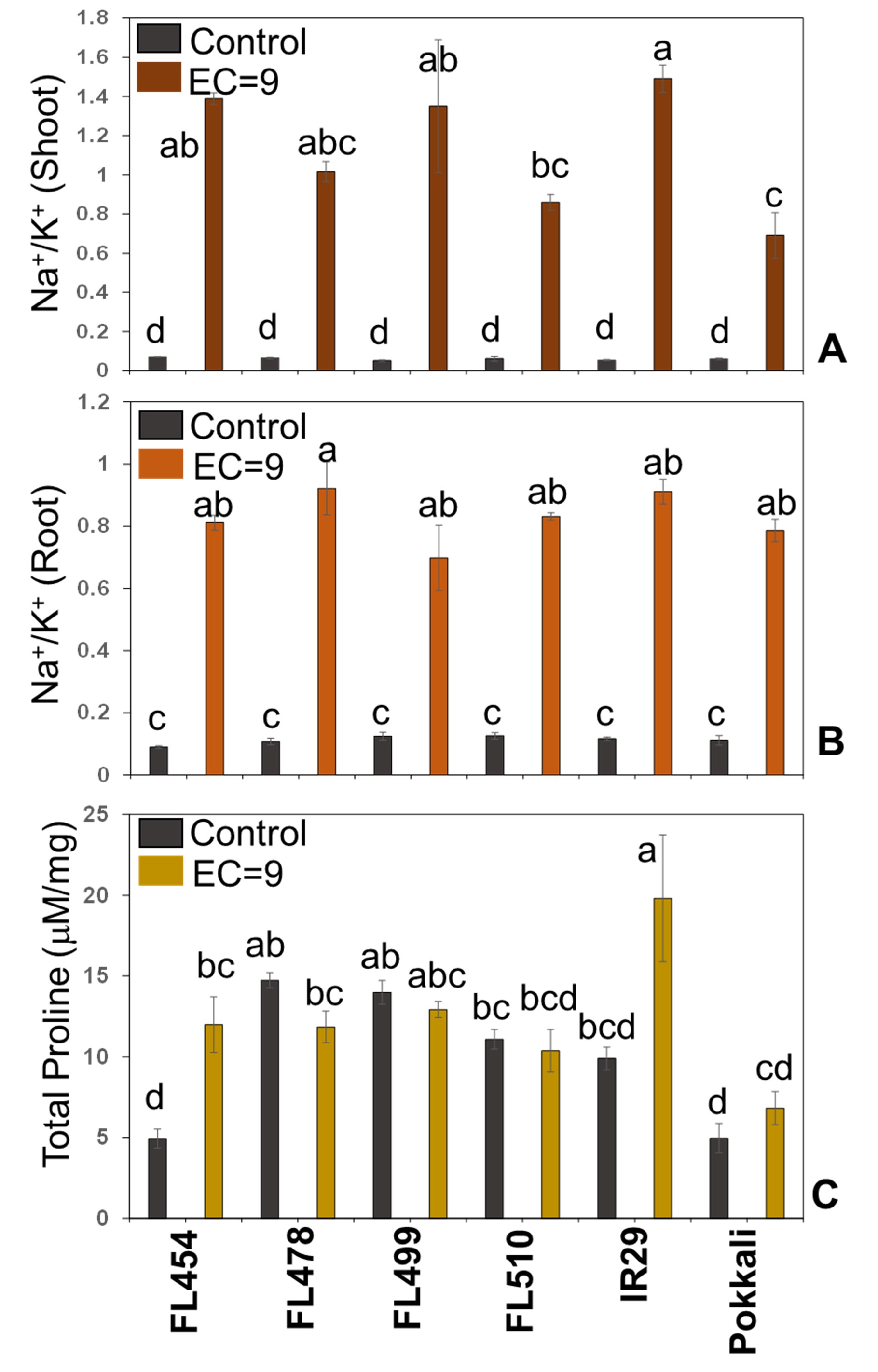

Supplement: Supplementary Figure 2 — Physiological characterization of the representative genotypes used in the real-time growth profiling at EC = 9 dS m–1. Physiological indicators of cellular defense and adjustment potentials during the 18-day period at EC = 9 were evaluated including Na+/K+ in the shoot (A), Na+/K+ in the root (B), and total proline content in leaves (C). For the Na+/K+ analysis (A,B), bar graphs represent the mean Na+/K+ (n = 5) with standard errors. One-way ANOVA with an HSD test (α = 0.05) was used to determine significant differences between genotypes and treatments. Lower-case letters signify treatment groups, separated by significant mean differences, with “a” representing the highest means. For the proline content analysis (C), bar graphs represent the means of total proline in leaves (n = 3) with standard error bars. One-way ANOVA with HSD test (α = 0.05) was used to determine significant differences between the different treatments. Lower-case letters signify treatment groups separated by significant mean differences, with “a” representing the highest means. [file Image_2.JPEG]

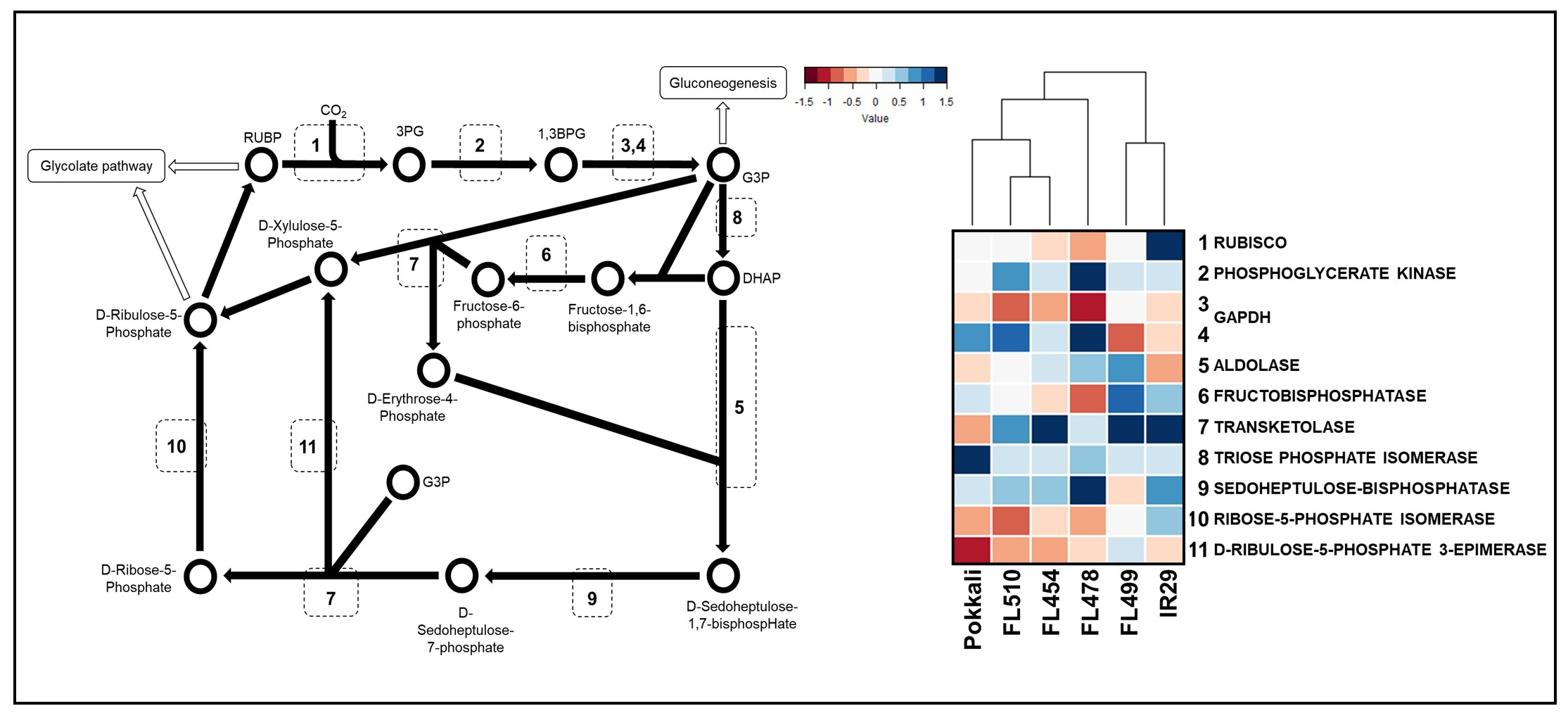

Supplement: Supplementary Figure 3 — Pathway induction heatmaps under stress for genes in the Calvin cycle. Values were generated from the fold-change under control conditions and the stress time point with the widest margin from control. The genotypes were hierarchically clustered to show similarity in pathway induction. RuBP, Ribulose 1,5-bisphosphate; RUBISCO, Ribulose-1,5-bisphosphate carboxylase/oxygenase; 1,3-BPG, 1,3-bisphosphoglycerate; PPK, Phosphoglycerate kinase; 3-PG, 3-phosphoglycerate; GAPDH, Glyceraldehyde-3-phosphate dehydrogenase; GAP, Glyceraldehyde-3-phosphate; TPI, Triose-phosphate isomerase; DHAP, Dihydroxyacetone phosphate; G3P, Glyceraldehyde 3-phosphate. [file Image_3.JPEG]
